# COVID-19-related hospital admission in spouses of partners in at-risk occupations

**DOI:** 10.5271/sjweh.4080

**Published:** 2023-03-30

**Authors:** Jens Peter Ellekilde Bonde, Luise Mølenberg Begtrup, David Coggon, Johan Høy Jensen, Esben Meulengracht Flachs, Kristina Jakobsson, Christel Nielsen, Kerstin Nilsson, Lars Rylander, Andreas Vilhelmsson, Kajsa Ugelvig Petersen, Sandra Søgaard Tøttenborg

**Affiliations:** 1 Department of Occupational and Environmental Medicine, Copenhagen University Hospital - Bispebjerg and Frederiksberg, Copenhagen, Denmark; 2 Department of Public Health, University of Copenhagen, Copenhagen, Denmark; 3 MRC Lifecourse Epidemiology Centre, University of Southampton, Southampton, UK; 4 School of Public Health and Community Medicine, Sahlgrenska Academy, University of Gothenburg, Sweden; 5 Division of Occupational and Environmental Medicine, Department of Laboratory Medicine, Lund University, Lund, Sweden; 6 Division of Public Health, Kristianstad University, Kristianstad, Sweden

**Keywords:** cohort study, family, industry, job, SARS-CoV-2, virus transmission, vulnerable group

## Abstract

**Objective:**

This study aimed to quantify the risk of COVID-19-related hospital admission in spouses living with partners in at-risk occupations in Denmark during 2020–21.

**Methods:**

Within a registry-based cohort of all Danish employees (N=2 451 542), we identified cohabiting couples, in which at least one member (spouse) held a job that according to a job exposure matrix entailed low risk of occupational exposure to SARS-CoV-2 (N=192 807 employees, 316 COVID-19 hospital admissions). Risk of COVID-19-related hospital admission in such spouses was assessed according to whether their partners were in jobs with low, intermediate or high risk for infection. Overall and sex-specific incidence rate ratios (IRR) of COVID-19-related hospital admission were computed by Poisson regression with adjustment for relevant covariates.

**Results:**

The risk of COVID-19-related hospital admission was increased among spouses with partners in high-risk occupations [adjusted IRR (IRR_adj_)1.59, 95% confidence interval (CI) 1.1–2.2], but not intermediate-risk occupations (IRR_adj_ 0.97 95% 0.8–1.3). IRR for having a partner in a high-risk job was elevated during the first three pandemic waves but not in the fourth (IRR_adj_ 0.48 95% CI 0.2–1.5). Sex did not modify the risk of hospital admission.

**Conclusions:**

SARS-CoV-2 transmission at the workplace may pose an increased risk of severe COVID-19 among spouses in low-risk jobs living with partners in high-risk jobs, which emphasizes the need for preventive measures at the workplace in future outbreaks of epidemic contagious disease. When available, effective vaccines seem essential.

SARS-CoV-2 (severe acute respiratory syndrome corona virus 2) infection has been recognized as an occupational hazard during the pandemic, and increased risk of COVID-19 has been reported in a number of occupations in several countries including Denmark (1–8). Besides the individual worker, family members may also become infected following secondary transmission of infection acquired in the workplace. Even though this aspect of occupational transmission of SARS-CoV-2 is recognized in the literature (9, 10), we have not identified any systematic study that quantifies the risk among family members of employees in at-risk occupations. The exception is a Swedish study indicating increased COVID-19 mortality among elderly individuals living together with employees with less opportunity to work from home during the pandemic (11).

SARS-CoV-2 exposure may cause asymptomatic but still communicable SARS-CoV-2 infection or clinical disease, which may be mild or severe. We use COVID-19-related hospital admission as a proxy for the latter and hereby give priority to study of a less frequent but serious outcome over more common asymptomatic SARS-CoV-2 infections and milder COVID-19 cases. Besides, the focus on COVID-19-related hospital admission is motivated by methodological issues because this outcome can be assumed to be independent of testing behavior which is an issue in studies based upon non-random SARS-CoV-2 test results (12, 13).

The objective of this paper was to examine the risk of COVID-19-related hospital admission among spouses of partners who were employed in documented intermediate- and high-risk occupations in Denmark.

## Methods

### Population and data

The source population was a nationwide cohort of all Danish employees aged 20–69 years (N=2 451 542) with public registry data on occupations and demographic, social and health characteristics including on COVID-19-related hospital admissions and COVID-19 vaccinations as detailed in an earlier paper (14). Occupations were those held on 31 December 2019, and were classified by the Danish version of the International Standard Classification of Occupations (DISCO-08) (15) at the 4-digit level (N=423 occupational groups).

### Occupational exposure classification.

Occupations were assigned to three levels of potential for exposure to SARS-CoV-2.

*Low-risk occupations* (N=50) were those which in an expert-rated job-exposure matrix (JEM) had a sum score of 0 across eight determinants of occupational SARS-CoV-2 exposure (possible range 0–24) (16).

*High-risk occupations* were specified for men and women separately, and defined as those which, in follow-up analyses through 2020–2021, relative to low-risk occupations, had an adjusted incidence rate ratio (IRR) >1.5 for hospital admission due to COVID-19, with a lower 95% confidence limit above unity (equivalent to a two-sided P<0.05) (4). These analyses were based upon the entire source population (N=2 451 542) and methods used to compute IRR estimates are detailed in an earlier publication (4). The criteria to define high-risk occupations were set *a priori* to balance the needs for magnitude and reliability of effects on one hand and sample size and statistical power on the other hand. High-risk occupations among men (N=19) and women (N=16) are listed in the supplementary material (https://www.sjweh.fi/article/4080), table S1.

*Intermediate-risk occupations* were all occupations not fulfilling the criteria for either high-risk or low-risk occupations (neither a reference occupation nor an occupation associated with elevated fully adjusted risk of COVID-19-related hospital admission).

### Study population

Within the source population, we identified cohabiting couples, comprising two adults, both aged 25–69 years with valid 4 digit-DISCO-08 codes, who met one of the following criteria: (i) married people (opposite-sex couples); (ii) people in a registered partnership (same-sex couples); (iii) two opposite-sex persons sharing residence with ≥1 shared child; (iv) two opposite-sex persons sharing residence with <15 years age difference without shared children and without sibling or parent-child relationship.

The study population (N=192 807) comprised those members of cohabiting couples, who were working in jobs with low risk of occupational exposure to SARS-CoV-2 infection. We refer to these individuals as *spouses*, and to the other members of the couples as the spouses’ *partners*. Partners could be working in occupations with low, intermediate or high risk of exposure to SARS-CoV-2. People < 25 years of age were excluded to ensure that the study population exclusively comprised couples of two adults (adult children <25 years of age living at home are included in the family definition used by Statistics Denmark).

**Figure 1:**
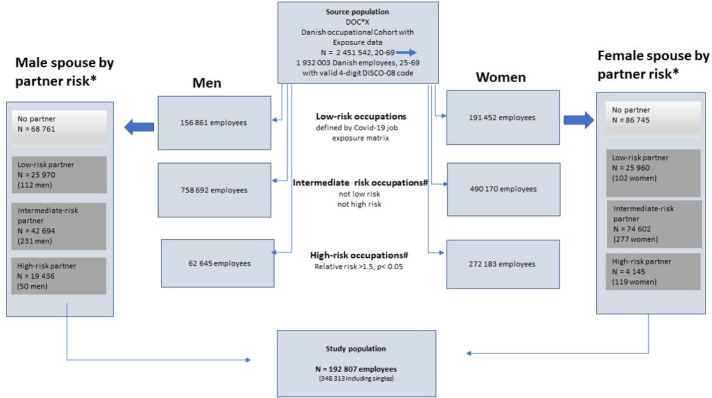
Identification of spouses (low-risk occupations) to partners in low-, intermediate- and high-risk occupations based upon the DOC*X cohort. * Including partners of same sex. # High-risk occupations were derived from sex-stratified follow-up analyses of adjusted risk of COVID-19 hospital admission (Poisson regression providing incidence rate ratios) by all 4-digit DISCO-08 job codes using the entire source population [N=2 451 542, results provided in supplementary table 1, methods detailed in (4)].

Employees in low-risk occupations who were not living with a spouse/partner according to the above definitions (N=155 506) were excluded from the main analyses but included in a sensitivity analysis (figure 1).

**Table 1:** Characteristics of male and female spouses according to risk of exposure to SARS-CoV-2 in job held by partner.

Characteristic	Male spouse in low-risk job			Female spouse in low-risk job		
	
	Partner in low-risk job N=25 970	Partner in intermediate-risk job N=42 694	Partner in high-risk job N=19 436	Partner in low-risk job N=25 960	Partner in intermediate-risk job N=74 602	Partner in high-risk job N=4145
					
	%	%	%	%	%	%
Age, years						
20–<30	7.1	7.0	5.7	10.4	8.8	6.6
30–<40	24.6	25.4	23.3	26.6	24.5	23.3
40–<50	30.3	30.9	31.4	31.2	29.9	28.3
50–<60	28.3	27.0	27.3	26.2	29.8	31.9
≥60	9.7	9.7	12.4	5.6	7.0	10.0
Geographical region						
Capital	43.4	40.1	28.8	43.3	32.9	33.1
Zealand	12.6	12.1	14.4	12.6	14.5	14.0
South	12.9	14.7	19.1	13.0	17.5	17.5
Central	23.2	24.0	26.3	23.4	24.0	22.3
North	7.9	9.2	11.5	7.9	11.1	13.2
Duration of education, years						
≤10	35.5	37.3	30.7	34.4	25.5	28.3
>10–13	47.8	45.3	50.9	52.8	59.5	56.3
>13–16	13.9	14.2	15.2	10.9	12.7	13.0
>16	1.9	2.3	2.7	1.0	1.7	1.7
Missing	0.9	0.9	0.5	0.8	0.6	0.7
Country of birth						
Denmark	92.7	92.2	94.6	90.6	92.3	90.4
Other western countries	3.5	3.1	1.9	3.7	2.6	2.7
Eastern Europe	1.8	1.7	1.0	3.2	2.8	3.1
Other countries	2.1	3.0	2.5	2.5	2.4	3.8
Number of hospital admissions 2010–2020						
0	86.7	86.6	85.2	81.8	78.9	78.2
1	11.1	11.2	12.6	15.8	17.7	18.3
≥2	2.2	2.2	2.2	2.5	3.4	3.5
Probability of tobacco smoking (JEM assigned)						
<10	5.3	4.2	4.1	6.1	4.3	3.4
10–<20	80.4	79.3	77.6	89.9	90.5	92.2
≥20	14.3	16.5	18.3	4.0	5.2	4.4
Bodymass index kg/m2 (JEM assigned)						
<25	10.1	9.4	7.6	51.6	43.7	41.5
≥25	89.9	90.6	92.4	48.4	56.3	58.5
Number of household members						
1	0	0	0	0	0	0
2	33.9	33.1	30.4	33.6	36.3	39.3
3	22.9	21.9	20.4	23.0	22.7	22.5
4	33.9	33.5	34.3	34.0	31.7	28.2
≥5	9.3	11.5	14.9	9.4	9.3	10.0
Number of children <15 years						
0	50.5	49.0	47.0	50.3	54.1	57.0
1	20.8	20.4	4.7	21.0	19.4	18.4
2	24.0	24.8	25.6	24.0	22.0	19.4
≥3	4.7	5.8	7.7	4.7	4.5	5.2
Second COVID-19 vaccination obtained						
1 January 2021 to 30 June 2021	20.8	20.2	22.0	15.1	16.7	20.2
1 July 2021 to 14 December 2021	75.6	76.0	74.1	79.7	77.5	73.6
<2 vaccinations by 14 December 2021	3.4	3.8	4.0	5.2	5.8	6.2

### Main exposure variable

The principal exposure variable was the risk of occupational exposure to SARS-CoV-2 in the partner’s job, with the low-risk category taken as the reference.

### Outcome

The outcome was severe COVID-19 defined by admission to hospital for a duration of ≥12 hours in combination with a positive PCR test within the 14 days before admission. This was ascertained from records in public registries hosted by Statistics Denmark and the Danish Health Data Authority by linkages using the Danish unique personal 10-digit identifier. During the study period, COVID-19-related hospital admissions were due to serious clinical COVID-19 in the vast majority of cases. However, according to ICD-10 diagnoses available from the National Patient Registry for a subset of the population, about 2.5% of cases were likely related to psychiatric, traumatic, or obstetric disorders.

### Covariates

Individual-level information on a range of demographic, social and health variables at the end of 2019 were obtained from public registries hosted by Statistics Denmark: sex, age, duration of education in years, country of origin, hospital admission for one or more of eleven chronic diseases during 2010–2019, geographical residential area and date of COVID-19 vaccinations. From data on household members including children, elders, and family members without gainful employment, we defined variables indicating the size of the household in terms of individuals sharing the same residence and the number of children <15 years old. Data on residential area per person were not available.

Estimates of the probability of current smoking and of body mass index (kg/m^2^) were assigned by lifestyle JEM based on questionnaire information from several large random samples of the Danish population representative for 2010 (17). The distribution of covariates across reference and high-risk exposure categories of study population spouses is displayed in table 1, which also shows the categorial grouping of covariates used in the statistical analyses

Further details on the cohort and its key variables are provided in an earlier paper (4).

### Statistical analysis

The study population was followed from week 8 in 2020 through to week 50 in 2021. This follow-up period was divided into four pandemic waves by midpoints of the troughs between peaks of COVID-19-related hospital admissions in Denmark. We used Poisson regression to compute overall and wave-specific IRR with 95% confidence intervals (CI) for COVID-19-related hospital admission. The time unit was a week, and follow-up was censored at the first COVID-19-related hospital admission, death, emigration, retirement or the end of week 50 in 2021. This approach is equivalent to Cox regression with an assumption of constant baseline risk in defined time periods.

Risk estimates were in accordance with the disjunctive confounder variable selection criteria adjusted by well-established determinants of the outcome without consideration of association with exposures (18): sex (in combined analysis only), age, duration of education, country of origin, geographical area, chronic disease, size of the household, body mass index, smoking and completed COVID-19 vaccination (two injections approximately 14 days apart). All covariates were determined at baseline except vaccination (which was treated as a time-varying variable). Moreover, estimates of risk across the entire follow-up period were adjusted for epidemic wave (also treated as a time-varying variable).

To assess if the pandemic wave and sex modified the risk of COVID-19-related hospital admission, we included interaction terms in separate regressions models (exposure×pandemic wave and exposure×sex, respectively).

In order to be comparable with spouses potentially at risk through their partners’ occupational exposure, the reference group for the main analysis included only employees in low-risk occupations living together with a partner in a low-risk occupation. To obtain a substantially larger reference group and increased statistical power, we augmented the reference group with singles in a sensitivity analysis.

All analyses were carried out in SAS 9.4 (SAS Institute, Cary, NC, USA) by remote and secured access to a platform at Statistics Denmark.

## Results

We observed in total 316 COVID-19-related hospital admissions among the 88 100 cohabitating men and 104 707 cohabitating women employed in one of 50 low-risk occupations through 18 148 351 person-weeks of follow-up. The overall adjusted risk was increased in employees whose partners held high-risk occupations, but not in employees with partners in intermediate-risk occupations. However, most CI were broad and included unity (table 2). Pandemic wave modified the risk associated with having a partner in an at-risk occupation (the P-value for interaction between exposure and pandemic wave in fully adjusted analyses was 0.10) (table 2). During the first three waves, risk was elevated among spouses with partners in high-risk jobs, while it was below unity in the last wave among both men and women (table 2). Sex did not modify the risk of hospital admission (P-value for interaction=0.87) and the sex-stratified results displayed in table 2 indicate similar risk patterns among men and women, although numbers are small in the stratum of female spouses with partners in high-risk occupations.

**Table 2:** Incidence rate ratios (IRR) with 95% confidence intervals (CI) for COVID-19 (C-19)-related hospital admission in spouses of partners with intermediate- and high-risk jobs ^1.^

		All waves			Wave 1 week 8–32 2020 Alpha variant dominates			Wave 2 week 33–52 2020 and 1–4 2021 Beta variant dominates			Wave 3 week 5–26 2021 Beta variant dominates			Wave 4 Week 27–50 2021 Delta variant dominates		
					
	N employees	N C-19	IRR ^2^	95% CI	N C-19	IRR2	95% CI	N C-19	IRR ^2^	95% CI	N C-19	IRR ^2^	95% CI	N C-19	IRR ^2^	95% CI
Male and female spouses																
Partner with high-risk job ^1^	23 581	64	1.59	1.1–2.2	12	1.92	0.8–4.4	34	1.77	1.1–2.9	<15	1.79	0.9–3.7	<5	0.48	0.2–1.5
Partner with intermediate-risk job	117 296	172	0.97	0.8–1.3	24	1.07	0.5–2.2	70	0.86	0.6–1.3	43	1.18	0.7–2.1	35	0.88	0.5–1.6
Referents (partner with low-risk job) ^3^	51 930	80	1.00		11	1.00		36	1.00		16	1.00		17	1.00	
Male spouses																
Partner with high-risk job ^1^	19 436	55	1.64	1.1–2.4	11	2.02	0.8–5.1	29	1.79	1.0–3.2	<15	1.54	0.7–3.6	<5	0.68	0.2–2.7
Partner with intermediate-risk job	42 694	74	1.03	0.7–1.5	13	1.02	0.4–2.5	31	0.95	0.5–1.7	15	0.88	0.4–2.0	15	1.52	0.6–3.9
Referents (partner with low-risk job) ^3^	25 970	44	1.00		8	1.00		20	1.00		10	1.00		6	1.00	
Female spouses																
Partner with high-risk job ^1^	4145	9	1.44	0.7–3.0	< 5	1.48	0.2–14	5	1.75	0.6–4.8	<5	2.01	0.4– 10	<5	0.59	0.1–4.6
Partner with intermediate-risk job	74 602	98	0.94	0.6–1.4	11	1.07	0.3–3.8	39	0.82	0.5–1.5	28	1.65	0.7–4.0	20	0.68	0.3–1.4
Referents (partner with low-risk job) ^3^	25 960	36	1.00		<5	1.00		16	1.00		<10	1.00		11	1.00	

1 Adjusted risk above 1.5 with a P-value < 0.05 in sex-stratified analyses of all occupations at the 4-digit DISCO-08 level (N=374 for men and N=348 for women).

2 Adjusted for sex, age (10 year groups), duration of education at baseline (5 groups), number of hospital admissions for one or more of 11 chronic diseases in the 10 years preceding start of the pandemic (3 groups), country of origin (4 groups), geographical region (5 groups), number of household members (5 groups), number of children < 15 years of age in the household (4 groups), probability of tobacco smoking (3 groups), bodymass index (2 groups) and completed COVID-19 vaccination (time varying variable, yes/no).

3 Employees with low likelihood of occupational SARS-CoV-2 exposure according to a COVID-19 job exposure matrix (sumscore for all eight rated indicators of SARS-CoV-2 workplace viral transmission = 0) (16).

Risk estimates were higher in sensitivity analyses that included low-risk employees without a partner in the reference group (303 cases among a total of 207 436 low-risk employees including singles, as opposed to 80 cases among 51 930 low-risk partners). Within the high-risk stratum, the combined risk for men and women increased from 1.59 to 1.77 (95% CI 1.3–2.4), among men from 1.64 to 1.66 (95% CI 1.2–2.3) and women from 1.44 to 1.80 (95% CI 0.9–3.6) (supplementary table S2).

## Discussion

In this follow-up study of COVID-19-related hospital admissions among Danish employees in low-risk occupations, we observed an overall elevated risk in the subset of spouses with partners working in high-risk occupations. The increase in risk vanished in the fourth wave of the pandemic (last half of 2021), which most likely reflects that 95.0% of the source population had completed vaccination by this time. Findings are compatible with a meta-analysis of contact-tracing studies showing a secondary household attack rate of 24% and a household reproduction number of 34% (19) and with a Canadian study of household infections associated with COVID-19 workplace outbreaks of (20).

### Limitations

The overlap between high-risk occupations among men and women was small. The risk was only elevated in both sexes in three of 35 occupations defined as high-risk occupations by the adopted criteria (see supplementary table S1). Therefore, analyses to define high- and intermediate-risk occupations were performed separately among men and women. In other words, an occupation defined as a high-risk occupation among men may not be a high-risk occupation among women and vice versa. Even though no obvious interaction by sex was observed, it should be acknowledged that few female spouses were at risk through their partners’ working in high-risk occupations, and that there is a risk of overlooking effect modification by sex.

Risk estimates were adjusted by ten established determinants of COVID-19-related hospital admission. These demographic, social and health characteristics were also in this cohort – with a few exceptions – strong predictors of the studied outcome [supplementary table S2 in reference (4)]. Domestic crowding is associated with risk of viral transmission (21, 22) and fewer opportunities for isolation at the home, but is, at least partially, accounted for by adjusting for social, ethnic and geographical characteristics including number of household members and children. Nevertheless, information about factors such as home-to-work commuting patterns (23, 24), large gathering attendance (25) and local hotspots (26) was not available at the individual level and skewed distribution of these factors across exposure categories may have contributed to residual confounding in either direction. The same applies to JEM-based assignment of probability of smoking and body mass index. Even these variables in this dataset have dose-related effects on COVID-19 hospital admission independent of other determinants (14), some residual confounding is likely – in part because the JEM based upon Danish surveys in 2010 and 2013 are slightly outdated.

It must also be acknowledged that the partner’s occupational risk is used as a proxy for domestic exposure. We do not know, if the individual employees in at-risk occupations actually had a SARS-CoV-2 infection in the relevant time window preceding the COVID-19-related hospitalization of the spouse. Although it seems plausible that partners, who are infected at the workplace, may indeed transmit the virus to the spouse and other family members at home, demonstration of pathways at the individual level would strengthen causal inference. Even though data on >40 million PCR tests conducted in the source population of this study are available, the test frequency in households in relevant time windows is far too limited to allow individual tracing.

While partners with intermediate- and high-risk jobs were excluded from the study population, couples in which both members held low-risk occupations contributed twice to the estimation of risk, violating the statistical requirement for independent observations. This is hardly an issue in the sex-stratified analyses because there were few same-sex couples, but it may have produced spuriously narrow CI in the combined analysis. Nevertheless, each partner in a couple was at risk of the outcome, and since none of the 79 hospital admissions in low-risk couples involved both partners, we believe that the possible impact on risk estimation is marginal.

The sensitivity analysis that broadened the reference group by including individuals in low-risk occupations living without an employed partner revealed higher risk estimates with narrower confidence intervals, reflecting the more than 3-fold larger reference-group. This gain in statistical power came at the cost of possible bias, as people living without a partner may have different social behaviors and risk profiles from cohabiting couples. However, the age- and sex adjusted risk for COVID-19-related hospital admission was only marginally lower in households with two people compared to singles (IRR 0.97 95% CI 0.89–1.07) in analyses based upon the entire source population (4). This indicates that it may be a minor issue in this population. Moreover, the sensitivity analysis including singles in the reference group was adjusted for size of the household.

### Implications

SARS-CoV-2 is a recognized occupational hazard in Denmark (27), but the present findings indicate, that occupational COVID-19 may reach beyond the individual employee. Workplace-related infection of the spouse and other family members is in particular of concern for vulnerable people such as the elderly and people with a range of chronic diseases (28, 29). This introduces a new perspective on management of occupational disease and adds to the importance of developing efficient preventive strategies, including for instance, consideration of strategies to mitigate domestic exposure and setting priorities for vaccination programmes (30).

### Concluding remarks

SARS-CoV-2 transmission in the workplace may expose spouses to higher risk of severe COVID-19, indicating a need for attention to preventive measures in the household as well as at the workplace in future outbreaks of epidemics of occupationally-transmitted contagious disease.

## Supplementary material

Supplementary material

## Data Availability

the pseudonymized database used for the presented analyses is hosted by Statistics Denmark and is not publicly available. Permission to access the database can be granted to researchers at research institution authorized by Statistics Denmark. On request the corresponding author can assist interested researchers to gain access.

## Permissions

Permissions to remotely retrieve, compile and analyze pseudonymized data at a secured and logged platform at Statistics Denmark were obtained from the Danish Data Protection Agency (P-2020-897), Statistics Denmark (P-708121) and the National Board of Health Data (FSEID-00005368). This process started in August 2020, and access to the data-files was provided in December 2021.

Ethical approval is by Danish law not required for studies that entirely are based on public registries.
