# Supplementary material for: COVID-19-related hospital admission in spouses of partners in at-risk occupations
Source: Scand J Work Environ Health. 2023 Mar 30;49(3):193–200. doi: 10.5271/sjweh.4080 (PMC10621900; doi:10.5271/sjweh.4080)
Supplement: Supplementary material [file SJWEH-49-193-S001.pdf]

COVID-19-related hospital admission in spouses of partners in at-risk occupations[1]

by Jens Peter Ellekilde Bonde, MD,[2] Luise Mølenberg Begtrup, PhD, David Coggon, DM, Johan Høy Jensen, PhD, Esben Meulengracht Flachs, PhD, Kristina Jakobsson, PhD, Christel Nielsen, PhD, Kerstin Nilsson, PhD, Lars Rylander, PhD, Andreas Vilhelmsson, PhD, Kajsa Ugelvig Petersen, PhD, Sandra Søgaard Tøttenborg, PhD

1. *Supplementary tables*
2. *Correspondence to: Jens Peter Bonde, Department of Occupational and Environmental Medicine, Bispebjerg and Frederiksberg Hospital, Bispebjerg Bakke 23, DK-Copenhagen 2400 NV, Denmark. [E-mail: Jens.Peter.Ellekilde.Bonde@regionh.dk]*

Table S1. High-risk 4-digit DISCO-04 occupational groups defined by an adjusted incidence rate ratio (IRR) above 1.5 and p-value < 0.05 compared to a reference group of low-level exposed employees[1] in a national cohort of employees aged 20-69. Stratification by sex.

Occupation (ISCO-08 label)	DISCO-08 code	Men		Women	
		N	IRR (95% CI)	N	IRR (95% CI)
Human Resource Managers	1212	1256	2.95 (1.5-6.0)		
Construction Managers	1323	3107	2.10 (1.2-3.7)		
Restaurant Managers	1412	440	4.12 (1.5-11)		
Generalist Medical Practitioners	2211	8423	2.04 (1.4-3.1)		
Specialist Medical Practitioners	2212			487	6.36 (2.3-18)
Nursing Professionals	2221	2536	1.96 (1.0-3.8)	60 054	2.10 (1.6-2.8)
Audiologists and Speech Therapists	2266	89	7.15 (1.0-51)		
Primary School Teachers	2341			51 275	1.51 (1.1-2.0)
Early Childhood Educators	2343			56 234	1.56 (1.2-2.0)
Other Music Teachers	2354			833	3.31 (1.0-10)
Other Arts Teachers	2355	55	9.18 (1.3-66)		
Teaching Professionals, Other	2359	763	2.95 (1.1-7.9)		
Psychological Therapists	2634	1768	2.37 (1.0-5.4)		
Translators and Interpreters	2643			823	4.81 (2.1-11.0)
Administrative and Executive Secretaries	3343	3684	2.15 (1.3-3.5)		
Social Work Associate Professionals	3412	1867	2.56 (1.4-4.6)		
Photographers	3431			235	7.31 (1.8-30)
Chefs	3434			1534	2.47 (1.0-6.0)
Building Caretakers	5153			1896	2.55 (1.2-5.5)
Nursing Aides	5321	9422	1.66 (1.2-2.4)	35 212	2.03 (1.6-2.6)
Home-Based Personal Care Workers	5322	8332	1.78 (1.2-2.6)	78 116	1.55 (1.2-1.9)
Air Conditioning and Refrigeration Mechanics	7127	1607	2.28 (1.0-5.1)		
Information and Communication Services	7422	489	3.79 (1.2-12)		
Dairy Product Makers	7513			857	3.19 (1.0-10)
Tobacco Prepares and Product Makers	7516			109	7.89 (1.1-57)
Sewing and Related Workers	7533	59	9.55 (1.3-69)		

Food Machine Operators	8160	12 184	1.75 (1.2-2.5)		
Packing, Bottling and Labelling Machine Operators	8183			1035	2.77 (1.1-6.8)
Bus Drivers	8331	9208	2.19 (1.6-2.9)		
Domestic Cleaners and Helpers	9111			665	4.64 (1.9-12)
Vehicle Cleaners	9122			165	7.89 (1.9-33)
Odd-Job Persons	9622	1022	2.59 (1.1-6.3)		

[1] Low likelihood of occupational SARS-CoV-2 exposure according to a population-based expert-rated job exposure matrix (16)

Table S2. Incidence rate ratio (IRR) with 95% confidence intervals (CI) for Covid-19 related hospital admission in spouses of partners with intermediate- and high-risk occupations[1]. Analyses including individuals without partners in the reference group.

		All waves			1. wave<br>week 8-32 2020<br>Alpha variant dominates			2. wave<br>week 33-52 2020, 1-4 2021<br>Beta variant dominates			3. wave<br>week 5-26 2021<br>Beta variant dominates			4. wave<br>Week 27-50 2021<br>Delta variant dominates		
	N employees	N Covid-19	IRR[2]	95% CI	N Covid-19	IRR[2]	95% CI	N Covid-19	IRR[2]	95% CI	N Covid-19	IRR[2]	95% CI	N Covid-19	IRR[2]	95% CI
Male and female spouses																
Partner with high-risk job[1]	23 581	64	1.77	1.3-2.4	<15	1.86	0.9-3.7	34	2.24	1.5-3.4	<15	1.90	1.0-3.5	<5	0.54	0.2-1.5
Partner with intermediate-risk job	117 296	172	1.12	0.9-1.4	24	0.98	0.6-1.7	70	1.18	0.9-1.6	43	1.20	0.8-1.8	35	1.01	0.7-1.6
Referents (No employed partner or partner with low-risk job)[3]	51 930	303	1.00	-	45	1.00	-	117	1.00	-	65	1.00	-	76	1.00	-
Male spouses																
Partner with high-risk job[1]	19 436	55	1.66	1.2-2.3	11	2.05	1.0-4.2	29	1.96	1.3-3.0	<15	2.09	1.1-4.1	<5	0.48	0.2-1.6
Partner with intermediate-risk job	42 694	74	1.04	0.8-1.4	13	1.05	0.5-2.1	31	1.00	0.7-1.5	15	1.19	0.6-2.2	15	1.10	0.6-2.0
Referents (No employed partner or partner with low-risk job)[3]	25 970	164	1.00	-	26	1.00	-	70	1.00	-	31	1.00	-	37	1.00	-
Female spouses																
Partner with high-risk job[1]	4145	9	1.80	0.9-3.6	< 5	1.66	0.2-13	5	2.90	1.1-7.4	<5	1.55	0.4-6.6	<5	0.70	0.1-5.2
Partner with intermediate-risk job	74 602	98	1.17	0.9-1.6	11	1.13	0.5-2.6	39	1.36	0.9-2.2	28	1.29	0.8-2.2	20	0.84	0.5-1.5
Referents (No employed partner or partner with low-risk job)[3]	25 960	139	1.00	-	19	1.00	-	47	1.00	-	34	1.00	-	39	1.00	-

[1] Adjusted risk above 1.5 with a p-value < 0.05 in sex-stratified analyses of all occupations at the 4-digit DISCO-08 level (n=374 for men and n=348 for women).

[2] Adjusted for sex, age (10 year groups), duration of education at baseline (5 groups), number of hospital admissions for one or more of 11 chronic diseases in the 10 years preceding start of the pandemic (3 groups), country of origin (4 groups), geographical region (5 groups), number of household members (5 groups), number of children < 15 years of age in the household (4 groups)probability of tobacco smoking (3 groups), bodymass index (2 groups) and completed Covid19 vaccination (time varying variable, yes/no).

[3] Employees with low likelihood of occupational SARS-CoV-2 exposure according to a Covid-19 job exposure matrix (sumscore for all eight rated indicators of SARS-CoV-2 workplace viral transmission = 0)(16) including same sex individuals without an employed partner.
